# Economic Effects of Introducing Alternative *Salmonella* Control Strategies in Sweden

**DOI:** 10.1371/journal.pone.0096446

**Published:** 2014-05-15

**Authors:** Kristian Sundström, Helene Wahlström, Sofie Ivarsson, Susanna Sternberg Lewerin

**Affiliations:** 1 Institute for Food and Agricultural Economics, Lund University, Lund, Sweden; 2 Zoonosis Center, National Veterinary Institute, Uppsala, Sweden; 3 Department of Preparedness, Swedish Institute for Communicable Disease Control, Solna, Sweden; 4 Department of Biomedical Sciences and Veterinary Public Health, Swedish University of Agricultural Sciences, Uppsala, Sweden; The Australian National University, Australia

## Abstract

The objective of the study was to analyse the economic effects of introducing alternative *Salmonella* control strategies in Sweden. Current control strategies in Denmark and the Netherlands were used as benchmarks. The true number of human *Salmonella* cases was estimated by reconstructing the reporting pyramids for the various scenarios. Costs were calculated for expected changes in human morbidity (*Salmonella* and two of its sequelae), for differences in the control programmes and for changes in cattle morbidity. The net effects (benefits minus costs) were negative in all scenarios (€ −5 to −105 million), implying that it would not be cost-effective to introduce alternative control strategies in Sweden. This result was mainly due to an expected increase in the incidence of *Salmonella* in humans (6035–57108 reported and unreported new cases/year), with expected additional costs of € 5–55 million. Other increased costs were due to expected higher incidences of sequelae (€ 3–49 million) and a higher cattle morbidity (€ 4–8 million). Benefits in terms of lower control costs amounted to € 4–7 million.

## Introduction

In addition to the common legislative framework for food safety in the European Union (EU), Sweden has a salmonella control programme that aims to ensure that domestic food of animal origin is entirely free of all *Salmonella*. Due to this, EU requirements for *Salmonella* control in poultry have been met for Swedish poultry long before joining the EU. A severe outbreak of *S*. Typhimurium in Sweden (SE) in 1953 that involved more than 9000 people prompted the need for a control programme for *Salmonella*. Since then, the strategy for control has been to prevent *Salmonella* in any part of the production chain. The current Swedish control programme covers the entire food chain from feed to food [1]. Any finding of *Salmonella* in animals, animal products or feed is notifiable according to the Swedish law on zoonoses (Zoonoslagen, SFS 2006∶1039), and measures to eliminate/eradicate *Salmonella* are taken at any positive finding. Restrictions are put on infected holdings until they can be declared free from *Salmonella*. The mandatory on-farm eradication is partly funded by the Board of Agriculture (with tax money) and the remaining cost is paid by the affected farmer. The level of co-funding varies with animal species and whether the farm is affiliated to a voluntary control programme. No funding is given for eradication on broiler holdings or beef herds buying animals from more than five sources. For other food-producing animals, 50% of documented costs are covered by the government, unless the farm is affiliated to a voluntary control programme, in which case up to 70% of documented costs may be covered by the government. The cost of HACCP controls in feed mills is paid by the feed producers (and thus, ultimately, the farmers). The costs for the control in animal products are paid for by the food industry.

A general estimation of the costs and the benefits of the SE *Salmonella* control was attempted in 1993 [2] but since then no analysis of the costs and benefits of the programme has been performed. As the national *Salmonella* programme has been in place for many years and a large amount of money is spent on it, an economic evaluation of the programme has been requested on several occasions. On EU-level, a consortium was recently commissioned to perform cost-benefit analyses of reducing the *Salmonella* prevalence in slaughter pigs and breeding pigs, respectively [3]. The results indicate large differences between countries in terms of the profitability of *Salmonella* control but also highlight a substantial lack of data. It is important to try to estimate the benefits and costs of reducing the *Salmonella* prevalence in countries with a high prevalence of *Salmonella* in food-producing animals. However, in countries where a well functioning and efficient *Salmonella* control is already in place, analysing a potential relaxation of the current control measures is more relevant. In 2011, based on the above, the SE *Salmonella* committee (an advisory committee consisting of representatives from competent authorities and the animal industry) commissioned the authors of this study to perform an evaluation of the costs and benefits of the SE control programme as compared to other less costly options. As it is not known what effect a change in different parts of the SE *Salmonella* control program would have on public health it was decided to compare the SE programme with other existing, less costly, control programmes. In [4], the expected increase in reported domestic human cases due to implementing two different alternative *Salmonella* control programmes in Sweden were estimated. The current paper estimates the underdetection and underreporting of *Salmonella* cases in SE and analyses possible economic effects of introducing these alternative *Salmonella* control strategies in SE.

## Material and Methods

The economic effects of a hypothetical implementation of the Danish and Dutch *Salmonella* control programmes in Sweden (hereafter called the DK and NL scenarios, respectively) were calculated. These calculations included costs for 1) an increasing number of domestic human cases of *Salmonella* and sequelae 2) changes in surveillance and control programmes and 3) production losses in cattle due to *S*. Dublin. The costs in 1) were based on estimations of expected increases in the number of *reported* domestic cases in four of the five different scenarios developed in [4]. In the current paper, these increases in the number of reported domestic cases were converted to expected increases in the *true* numbers of cases by a reconstruction of the reporting pyramid in each scenario. The methodologies involved in this reconstruction are discussed in more detail below.

### Estimation of the change in the number of reported domestic human cases

Changes in the expected number of reported domestic *Salmonella* cases in SE were estimated in [4] using five different data sources and two different baseline countries (DK and NL). These calculations were based on the assumption that the domestic *Salmonella* incidence in SE would become equal to the incidences in DK and NL, respectively, based on the five different data sources. As discussed by the authors, one of these data sources (based on travel data), yielded unrealistically high estimates and has thus been excluded from the cost analysis in the current paper.

### Estimation of the true number of domestic human cases of salmonellosis and its sequelae

Due to underreporting and underdetection, the number of reported *Salmonella* cases only constitutes a minor part of all clinical *Salmonella* cases. All *Salmonella* cases that have some degree of clinical symptoms (hereafter called salmonellosis cases) may lead to direct and indirect costs. In order to estimate the true number of salmonellosis cases, five different stages, at which information regarding a salmonellosis case might get lost, were identified: (1) no medical care is sought, (2) sample is not taken, (3) sample is not analysed for *Salmonella*, (4) non-perfect test sensitivity and (5) positive test is not reported. In addition, two factors were identified that affects the probabilities of information losses; 1) having bloody diarrhoea was assumed to affect the probability of a) seeking care and b) having stool samples taken, and 2) the type of unit (GP or hospital) carrying out the examination was assumed to impact the probability of stool samples a) being taken and b) being analysed for *Salmonella*. The probabilities of having information losses at any of these five different stages were modelled by betapert distributions with parameter values based on expert opinion (see Appendix S1) and data from a recent study on the incidences of seven different pathogens in the EU [5]. The risk of getting bloody diarrhoea was modelled by a beta distribution, which was fit to available outbreak data from various countries (mostly from the U.S.) between 1995 and 2009 (see Appendix S5).

To enable an estimation of the costs of illness, the salmonellosis cases were also partitioned into four different outcome classes (no care, GP only, hospitalization and death). Data regarding reported hospitalized cases (2010) and deaths (1998–2008) were obtained from databases at the National Board of Health and Welfare in Sweden. The proportions of salmonellosis cases allocated to each of these outcome classes were calculated using the model described in Appendix S1. Apart from applying different direct costs to the different outcome classes, the number of days of illness was also assumed to differ depending on outcome class (see Appendix S2 for details), affecting the size of productivity losses per case of each outcome class.

Two sequelae of salmonellosis were accounted for in this study: Irritable Bowel Syndrome (IBS) and Reactive Arthritis (ReA). Because of a high background prevalence of IBS, attributable risk (AR) (where risk for controls is subtracted from risk for cases) was used to estimate the expected proportion of salmonellosis cases leading to post-infection (PI) IBS (see Appendix S4). The estimation of AR was based on five studies [6]–[10], which were selected based on a meta-analysis [11] which was discussed and extended in a NL study on the economic effects of PI-IBS [12]. The proportion of salmonellosis cases leading to PI-ReA was estimated based on the literature (see Appendix S3).

The total number of cases of either sequelae was then partitioned into the three different outcome classes (no care, GP only and hospitalization). Since neither of these illnesses has been associated with mortality, ‘death’ was excluded from the possible outcome sets. For PI-IBS, the proportions of cases seeking care were based on a SE population study [13]. The proportion of cases that was hospitalized was estimated based on a survey-based American study [14] under the assumption that there would only be one visit each year for each IBS patient. The proportions of PI-ReA cases in the three different outcome classes were estimated based on the results in a Finnish study on campylobacter-triggered ReA [15].

### Assessment of the economic impact of domestic salmonellosis cases and their sequelae

The total costs for a change in the number of salmonellosis cases were calculated as the sum of 1) direct costs, 2) indirect costs and 3) intangible costs. Firstly, direct costs (*i.e.* costs that arise as a direct consequence of an illness) included costs for medication, GP visits, hospitalizations and transports to and from care units. The data sources used to estimate these direct costs are specified in Appendix S2. Secondly, indirect costs (*i.e.* production losses that arise as a consequence of sick leave) were estimated as the net salaries for all salmonellosis cases (as well as for the parents of child cases) during the sick leave period according to neo-classical wage theory [16]. Two alternative methods for estimating indirect costs were used, the friction cost method, using a friction period of 90 days, and the human capital method. Fundamentally, these two methods differ in terms of how they view the inner workings of the labour market, and, as a consequence, the maximum length of the sick leave period to account for in the cost estimations. Thirdly, intangible costs include valuations of immaterial aspects of an illness like pain, unease, nausea and sorrow due to either non-fatal outcomes (Value of a Statistical Case, VSC) or deaths (Value of Statistical Life, VSL). In this paper we included VSL but not VSC in the cost estimations for salmonellosis. For the VSL estimations, we utilized a value of € 2.5 million, which has been estimated in and is used by the transport sector in Sweden [17]. Using life expectancy data in conjunction with data for age and gender of the respondents in the above study [17], the VSL value was converted to a value for a lost life-year (VSLY) amounting to € 66000. This value was then applied to the specific distributions of age and gender of registered death cases due to salmonellosis to estimate the intangible costs. Direct costs and indirect costs were applied on PI-IBS and PI-ReA in the same manner as for salmonellosis. Since ‘death’ was excluded from the possible outcome set, intangible costs in terms of VSL were equal to zero for these sequelae.

### Stochastic simulation

To account for the variability and uncertainty associated with estimating the true number of salmonellosis cases as well as the costs related to these cases, Monte Carlo simulations using 100000 iterations were carried out, using the RiskAMP Monte Carlo Add-in for Excel. All distributions and point estimates included in these simulations are detailed in Appendix S1-S5.

### Costs for the current SE control programme

The current programme includes surveillance in the entire chain from feed to food, and eradication measures whenever *Salmonella* is found in feed mills, or on farms. Some of these costs are borne by the producers and some are paid by the government (see Table 1). To estimate the costs for the current on-farm eradication measures of *Salmonella* in food-producing animals, figures were obtained from the department for animal welfare and health in the Board of Agriculture. As the cost for on-farm eradication is shared by the farmer and the Board of Agriculture, with the latter paying for 50–70%, the registered government costs did not reflect the full cost. To rectify this, the money paid to animal owners for on-farm eradication activities were re-calculated to 100%, for the herds with 50% and 70% co-financing, respectively. These sums were used for estimating the total direct costs for on-farm eradication. In addition, the costs for the official veterinarians responsible for each positive farm and the laboratory costs for samples taken during the eradication process were also included in the direct costs. To reduce the effect of yearly variations, an average cost per year, based on figures from the last six years (2005–2010), was used. However, remaining payments (*i.e.* outstanding claims received within the period 2005–2010) for the feed-borne outbreak of *Salmonella* Cubana in 2003 [18] were not included. The reason for this is that this particular outbreak included exceptional cleaning and disinfection measures in the entire feeding systems of many large pig herds and may therefore not be representative of the eradication costs. Based on the experiences from this outbreak, new guidelines for cleaning and disinfection of feeding systems have been developed, and present and future eradication costs are thus not comparable with the 2003 outbreak.

**Table 1:** Detailed cost estimates for *Salmonella* surveillance and control in feed, animals and food (current cost and costs for systems similar to the ones in DK and NL, respectively).

Type of cost	Current cost	Cost in DK scenario	Cost in NL scenario
*Salmonella* eradication in infected cattle farms*	1405	0	0
*Salmonella* eradication in infected pig farms	498	0	0
*Salmonella* eradication in infected poultry farms	1388	1388	1388
Industry's cost for surveillance and control in cattle	587	701 (418)	254 (126)
Industry's production losses due to *Salmonella* Dublin in cattle	0	7852 (3926)	7852 (3926)
Industry's cost for surveillance and control in pigs	204	2817 (140)	423 (204)
Industry's cost for surveillance and control in poultry	1539	1539	1539
Cost for feed producers	4695	23	0
Surveillance in food items including slaughterhouses and cutting plants	1197	1128	1128
Total cost	11513	15448 (8562)	12584(8311)

Data sources, see text. All costs given in thousands of euros. Figures in brackets represent the estimates with the lower figures for laboratory costs and dairy production losses, respectively.

*Sum represents average per year based on figures from 2005–2010.

The total costs for the current compulsory and voluntary control programmes run by the industry were obtained from each organisation, respectively. Detailed figures were provided by the Swedish Dairy Association, the Swedish Animal Health Services the Swedish Poultry Meat Association and the Swedish Egg and Poultry Association.

The current costs for surveillance in slaughterhouses and cutting plants, and for extra import controls of food items (*i.e.* those items without certification of *Salmonella* freedom according to the SE *Salmonella* guarantees) were obtained from the financial department of the National Food Agency.

The costs for surveillance and control of *Salmonella* in the SE feed industry have been previously estimated [19]. Heat treatment of poultry feed according to EU legislation would remain, but this cost could not be separated from the other costs and was consequently included in the total cost that was regarded as entirely linked to the current control. It was assumed that all these costs would cease if the current *Salmonella* control programme would be abandoned. Detailed cost estimates are provided in Table 1.

### Costs for alternative control programs

In the DK and NL scenarios, the costs for the current control programme would be replaced by the costs for alternative control strategies. These were assumed to be similar to the current strategies in DK and NL, respectively. Briefly, surveillance would be conducted in pigs and cattle but on-farm measures would be voluntary. The costs for these measures were not possible to estimate and were therefore not included. In the DK scenario, some surveillance in feed was assumed and in both scenarios the control in poultry was assumed to be the same as in the current programme. Control in the food chain was assumed to follow EU legislation.

If these alternative costs are lower than the current costs, the result is a benefit.

Surveillance for *Salmonella* in poultry must be conducted according to EU legislation. These requirements match the current SE control programme for poultry, for the *Salmonella* serotypes most commonly found in poultry. Therefore, the cost of *Salmonella* control in poultry herds was assumed to remain the same if the current national control programme was to discontinue. However, the heat treatment of poultry feed in SE could not be separated from other costs for production of *Salmonella*-free feed (see above), and these additional costs were therefore not included.

The costs for a control programme for pigs and cattle according to the current practice in DK and NL, respectively, were re-calculated using the SE animal population. The costs for the DK feed control were also included in the DK scenarios. The costs for laboratory analyses were calculated based on the current prices in the national laboratories, and a discount was only assumed in case of a very large number of samples. Labour costs for sampling and sending samples were not included in the calculations, due to the difficulty of estimating these costs. Costs of € 12 for each serological analysis of serum or milk samples and € 23 for each culture sample were assumed in the DK scenarios. In the NL scenarios, where the total number of samples were fewer and thus no discount on laboratory prices or postage was assumed, € 14 for a serum/milk sample and € 35 for a culture sample were used in the calculations. These costs match the current SE prices for *Salmonella* analyses and postage. To assess the sensitivity of the final estimate of the cost for laboratory analyses, the calculations were also performed with assumed prices of € 7 for serology (serum/milk) and € 17 for bacteriology.

Information about the NL surveillance for the relevant time period was provided by GD Animal Health, Deventer, NL. Information about the DK surveillance was obtained from reports publicly available [20]. In short, in the NL scenario, it was assumed that pig herds were monitored by bacteriology (carcass swabs) at slaughter, with larger slaughterhouses submitting 10 samples/week and smaller ones 2 samples/week. This corresponds to the national scheme; slaughterhouses with US certification have other schemes but this was not included in the calculations. For cattle, three annual bulk tank milk samplings according to the national requirements of the dairy industry were assumed. In the DK scenarios, monitoring of pig herds was assumed to correspond to 10 monthly serum samples in breeding herds and five monthly meat juice samples at slaughter from fattening herds. In cattle, the assumed surveillance corresponded to four annual bulk tank milk samples/year in all dairy herds and three serum samples/year in beef herds. For the DK scenarios, monitoring of feed by 1000 samples/year was also included.

Sampling in the food chain without the current control programme would be based on the microbiological criteria as stated in the EU legislation. The National Food Agency provided estimates for sampling necessary to fulfil the EU requirements. Detailed cost estimates are provided in Table 1.

### Costs for the expected change in prevalence of Salmonella Dublin in cattle in the DK and NL scenarios

Production losses due to *S.* Dublin if the current control was to be discontinued have been previously estimated [21]. The figure for the low estimate for *S*. Dublin prevalence in this report, 10%, was used in the current study. This level was regarded as realistic as it corresponds to the situation in the region with the highest prevalence in Sweden. Detailed cost estimates are provided in Table 1. In order to test the sensitivity of the final estimate to this cost, halving the estimated cost for production losses was also tested in the calculations.

## Results

### Estimation of expected changes in the true number of domestic salmonellosis cases and their sequelae

**Figure 1:**
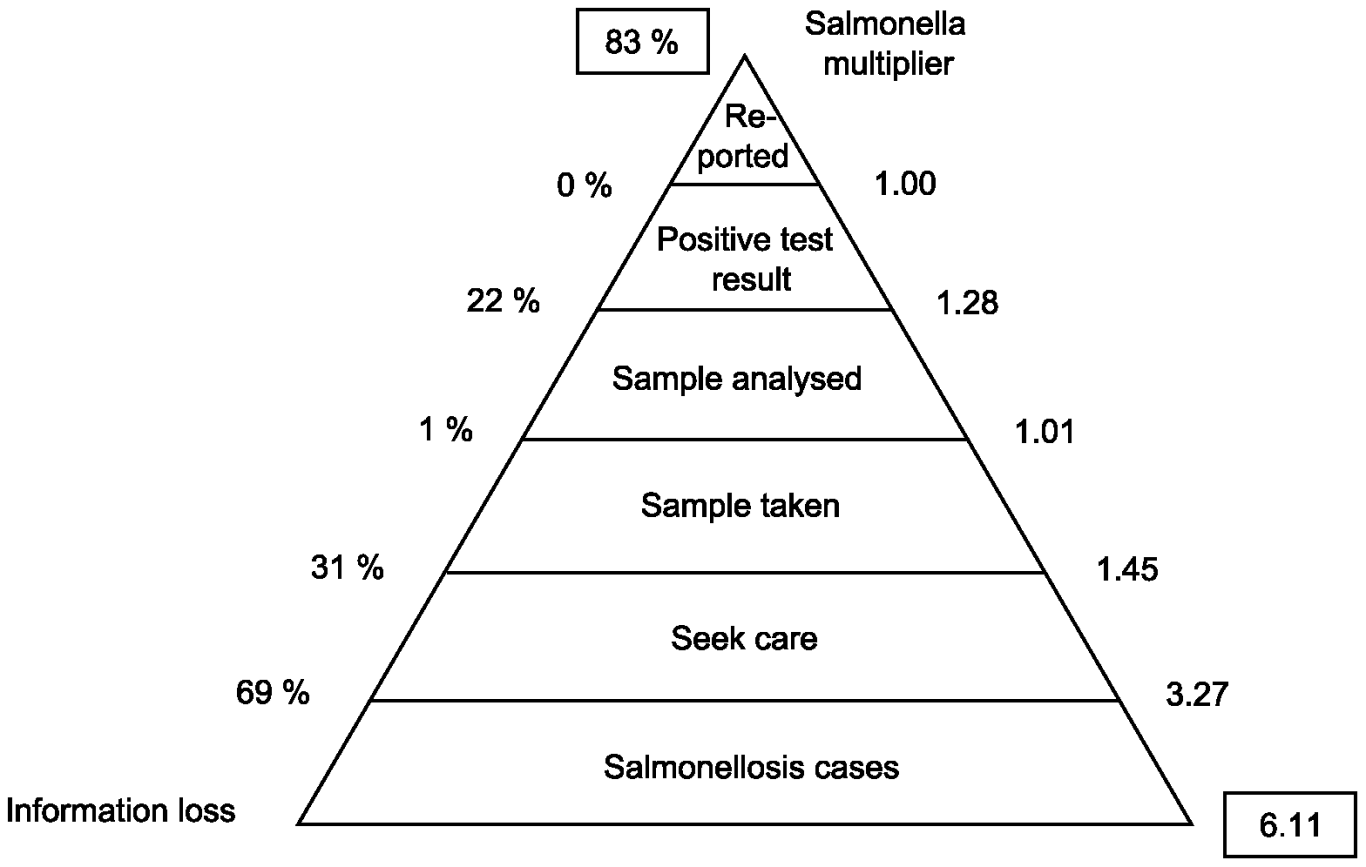
Reporting pyramid for salmonellosis.

In Figure 1, sequential multipliers for the five different stages of information loss for human salmonellosis as discussed above are provided as means from the simulations. Multiplying these five sequential multipliers with each other, results in an aggregated multiplier of 6.75, which indicates that in SE, the number of reported cases should on average be multiplied by 6.75 to obtain the true number of salmonellosis cases in the population. This corresponds to a probability of about 85% that a salmonellosis case will not be reported. The largest information losses are due to salmonellosis cases not seeking care (71%), samples not being taken of salmonellosis cases seeking care (33%) and imperfect test sensitivity (22%).

Combining estimates of the number of reported cases from [4] with the stochastic aggregate multiplier results in estimates of the increase in the *true* number of salmonellosis cases. Using this procedure, the mean true number of salmonellosis cases in SE, *i.e.* after corrections for underdiagnosis and underreporting, was estimated to increase with between 6035 and 15782 for the DK scenarios and between 42888 and 57108 for the NL scenarios (Table 2). The proportions of salmonellosis cases triggering IBS and ReA were estimated to 0.09 (c.i. 90% 0.07–0.11) and 0.08 (c.i. 90% 0.04–0.12), respectively. The increase in the number of cases of PI-IBS varied between 536 and 1402 for the DK scenarios, and between 3807 and 5076 for the NL scenarios (means). Correspondingly, the increase in the number of cases of PI-ReA was estimated to vary between 496 and 1288 for the DK scenarios, and between 3522 and 4695 for the NL scenarios (means from simulations).

**Table 2:** Estimated increase in the number of reported domestic human *Salmonella* cases, the true number of domestic human cases of salmonellosis, post-infectious ReA (PI-ReA) and post-infectious IBS (PI-IBS) if introducing alternative *Salmonella* control strategies.

	Estimated increase (c.i. 90%)			
Scenario Country/Method*	in reported domestic *Salmonella* cases	in true number of salmonellosis cases	in true number of PI-ReA cases	in true number of PI-IBS cases
DK/1a	2351	15782 (6092–35979)	1288 (400–3105)	1402 (528–3216)
NL/1a	8404	57108 (21691–132398)	4695 (1421–11478)	5076 (1870–11737)
DK/1b	2222	14979 (5678–24037)	1227 (372–2960)	1330 (491–3063)
NL/1b	6667	45023 (17099–102631)	3696 (1119–8927)	3996 (1475–9157)
DK/2a	1822	12202 (4694–27403)	1002 (306–2366)	1084 (402–2468)
NL/2a	8124	55216 (20891–125087)	4515 (1356–10859)	4898 (1800–11290)
DK/2b	1258	8497 (3226–19395)	698 (212–1693)	755 (279–1739)
NL/2b	6356	42888 (16367–97455)	3522 (1066–8525)	3807 (1407–8709)
DK/4a	1674	11193 (4293–25486)	920 (278–2216)	995 (368–2258)
DK/4b	987	6672 (2534–15181)	547 (166–1313)	593 (218–1364)
DK/5a	1623	10968 (4169–24994)	900 (273–2180)	973 (360–2231)
DK/5b	894	6035 (2298–13731)	496 (150–1194)	536 (199–1231)

Means and credibility intervals based on 100 000 Monte Carlo simulations.

*Indicates method used in [4] to estimate an increase in the number of reported domestic *Salmonella* cases. 1: Sero-Incidence, 2: Travel Data I, 4: Reconstruction of the reporting pyramid, 5: Expert opinion.

### Estimation of increased costs for salmonellosis cases and their sequelae

The increased costs for the true number of salmonellosis cases and their sequelae for the different scenarios are summarized in Table 3. For the DK scenarios, the increase in the total mean costs amount to between € 8–21 million (friction cost method) and € 11–29 million (human capital method). Corresponding mean costs for the NL scenarios are between € 57–76 million (friction cost method) and € 78–104 million (human capital method). Depending on the method used to estimate indirect costs, salmonellosis thus constituted between 52% and 68% of public health costs, while the proportion of this total cost due to PI-ReA and PI-IBS varied between 7% and 10%, and between 24% and 41%, respectively.

**Table 3:** Costs (millions of euros) due to the estimated increase in the number of domestic human salmonellosis cases if introducing alternative control strategies.

Scenario Country/Method*	Method indirect costs	salmonellosis	PI-ReA	PI-IBS	Total
DK/1a	FCM	14 (8–27)	2 (1–5)	5 (1–12)	21 (10–42)
	HCM	15 (8–28)	2(1–5)	12 (3–29)	29 (13–60)
NL/1a	FCM	52 (29–97)	7 (2–18)	17 (5–43)	76 (38–153)
	HCM	55 (30–103)	7 (2–18)	42 (12–104)	104 (47–218)
DK/1b	FCM	13 (7–25)	2 (0–5)	5 (1–11)	20 (10–40)
	HCM	14 (8–27)	2 (0–5)	11 (3–27)	27 (12–57)
NL/1b	FCM	41 (23–76)	6 (1–14)	14 (4–34)	60 (30–120)
	HCM	43 (24–81)	6 (1–14)	33 (9–82)	81 (37–171)
DK/2a	FCM	11 (6–21)	2 (0–4)	4 (1–9)	16 (8–33)
	HCM	12 (6–22)	2 (0–4)	9 (2–23)	22 (10–47)
NL/2a	FCM	50 (28–93)	7 (2–17)	17 (5–41)	73 (37–146)
	HCM	53 (29–99)	7 (2–17)	41 (11–104)	100 (45–214)
DK/2b	FCM	8 (4–14)	1 (0–3)	3 (1–6)	11 (5–23)
	HCM	8 (4–15)	1 (0–3)	6 (2–16)	15 (7–32)
NL/2b	FCM	39 (22-73)	5 (1–14)	13 (4–32)	57 (28–115)
	HCM	41 (22–77)	5 (1–14)	31 (9–78)	78 (35–163)
DK/4a	FCM	10 (6–19)	1 (0–4)	3 (1–9)	15 (7–30)
	HCM	11 (6–20)	1 (0–4)	8 (2–21)	20 (9–43)
DK/4b	FCM	6 (3–11)	1 (0–2)	2 (1–5)	9 (4–18)
	HCM	6 (3–12)	1 (0–2)	5 (1–12)	12 (5–25)
DK/5a	FCM	10 (5–18)	1 (0–3)	3 (1–8)	14 (7–29)
	HCM	10 (6–20)	1 (0–3)	8 (2–20)	20 (9–42)
DK/5b	FCM	5 (3–10)	1 (0–2)	2 (1–5)	8 (4–16)
	HCM	6 (3–11)	1 (0–2)	4 (1–11)	11 (5–23)

Estimations made using the human capital method (HCM) and the friction cost method (FCM), respectively. Mean values and 90% credibility intervals.

*Indicates method used in [4] to estimate an increase in the number of reported domestic *Salmonella* cases. 1: Sero-Incidence, 2: Travel Data I, 4: Reconstruction of the reporting pyramid, 5: Expert opinion. Method 3 was excluded since it produced unrealistic results.

### Costs for the current SE control programme

The current costs include surveillance costs in food production, costs for on-farm eradication, costs for the voluntary surveillance and control programmes and surveillance and eradication costs for feed producers. These costs would cease if the DK or NL scenarios were implemented (Table 1). The total costs for the on-farm eradication of *Salmonella* in cattle, pigs and poultry were estimated to € 3.3 million per year. The programmes managed by the cattle, pig and poultry industries were estimated to € 2.3 million. The cost of the current control of *Salmonella* in feed was estimated to € 4.7 million per year and the current control in the food chain was estimated to € 1.2 million.

### Costs for alternative control strategies

The changed costs for the *Salmonella* surveillance in the DK and NL scenarios are equal to the costs listed here minus the cost for the current control. This is summarized in Table 1 and Table 4. The cost for *Salmonella* control in poultry according to EU legislation was estimated to € 1.5 million/year. The eradication costs in infected poultry flocks were estimated to remain at € 1.4 million, due to EU legislation. The cost for surveillance in cattle and pigs according to the current system in NL was estimated to € 0.7 million. Assuming the lower costs for laboratory analyses, the estimate was reduced to € 330000. The cost for surveillance in cattle, pigs and feed according to the DK programme was estimated to € 3.5 million. With the lower costs for laboratory analyses the estimate was reduced to € 574900. The estimated sampling to fulfil EU requirements for food was estimated to 30000 samples plus random sampling of 20% of imported consignments, corresponding to a total cost of € 1.1 million.

**Table 4:** Benefits, costs and net effects of introducing alternative control strategies for *Salmonella* in Sweden.

Scenario Country/Method*	Benefits**	Costs***	Net effect (benefits –costs)
DK/1a	Lower control costs: 3.9 (6.9)	Higher human illness costs: 21–29	−18 to −33
		Higher cattle morbidity costs: 7.9 (3.9)	
NL/1a	Lower control costs: 6.8 (7.1)	Higher human illness costs: 76–104	−73 to −105
		Higher cattle morbidity costs: 7.9 (3.9)	
DK/1b	Lower control costs: 3.9 (6.9)	Higher human illness costs: 20–27	−17 to −31
		Higher cattle morbidity costs: 7.9 (3.9)	
NL/1b	Lower control costs: 6.8 (7.1)	Higher human illness costs: 60–81	−57 to −82
		Higher cattle morbidity costs: 7.9 (3.9)	
DK/2a	Lower control costs: 3.9 (6.9)	Higher human illness costs: 16–22	−13 to −26
		Higher cattle morbidity costs: 7.9 (3.9)	
NL/2a	Lower control costs: 6.8 (7.1)	Higher human illness costs: 73–100	−70 to −101
		Higher cattle morbidity costs: 7.9 (3.9)	
DK/2b	Lower control costs: 3.9 (6.9)	Higher human illness costs: 11–15	−8 to −19
		Higher cattle morbidity costs: 7.9 (3.9)	
NL/2b	Lower control costs: 6.8 (7.1)	Higher human illness costs: 57–78	−54 to −79
		Higher cattle morbidity costs: 7.9 (3.9)	
DK/4a	Lower control costs: 3.9 (6.9)	Higher human illness costs: 15–20	−12 to −24
		Higher cattle morbidity costs: 7.9 (3.9)	
DK/4b	Lower control costs: 3.9 (6.9)	Higher human illness costs: 9–12	−6 to −16
		Higher cattle morbidity costs: 7.9 (3.9)	
DK/5a	Lower control costs: 3.9 (6.9)	Higher human illness costs: 14–20	−11 to −24
		Higher cattle morbidity costs: 7.9 (3.9)	
DK/5b	Lower control costs: 3.9 (6.9)	Higher human illness costs: 8–11	−5 to −15
		Higher cattle morbidity costs: 7.9 (3.9)	

Human illness and net effects expressed as means, other benefits and costs expressed as point estimates. All costs and benefits are given in millions of euros.

*Indicates method used in [4] to estimate an increase in the number of reported domestic *Salmonella* cases. 1: Sero-Incidence, 2: Travel Data I, 4: Reconstruction of the reporting pyramid, 5: Expert opinion.

**Calculated as control costs of alternative control strategies – control costs for current control. Figures in brackets represent cost if cheaper lab analyses are assumed.

***Figures in brackets represent cost if a lower change in dairy production losses is assumed.

In Table 4, the net effects (benefits minus costs) of introducing alternative *Salmonella* control strategies in SE are summarized. In all scenarios, the control costs for the current SE control were higher than the costs for the alternative control strategies, entailing that the combined effects (cost for current SE control minus control costs of alternative strategies) are all positive. This combined effect thus represents a benefit of introducing alternative control strategies (see Table 4).

### Increased costs for Salmonella Dublin in cattle without the current control programme

This is a cost that would only be present in the DK and NL scenarios, as it represents a change from the current situation (see Table 1). The direct costs due to production losses in infected herds were estimated to € 4.3 million. The secondary costs in the dairy and meat industries etc. were, for the same prevalence level, estimated to € 3.6 million. Assuming this was an overestimate and the increased losses would only be half of these estimates, the estimated total losses for the dairy industry would be € 4 million.

## Discussion

In all scenarios, the total net effects of introducing alternative control strategies are negative (€ −5 to −105 million, see Table 4). This means that it would not be cost-effective to exchange the current SE *Salmonella* control programme under any of the analysed scenarios. The estimated increases in reported domestic cases in SE in all scenarios are based on a previous study [4]. As available official statistics on the number of *Salmonella* cases in different countries were not comparable; the authors use data from five sources to estimate the true domestic incidence in SE, DK and NL in 2010. Using these estimates, the expected increase in reported domestic human cases in Sweden in the different scenarios were estimated by the authors. The differences in the five scenarios reflect the large amount of uncertainty associated with these estimates. However, apart from one of the data sources, which was excluded from the current study based on a discussion in [4], the results for DK are of the same magnitude. Figures for the NL scenarios vary more and an implementation of the NL scenarios would increase public health costs considerably more than the DK scenarios in most cases. The authors concluded that this highlights the need for truly comparable data. In line with the above observations in [4], it can be concluded in the current study that in the DK scenarios, the costs are expected to increase about € 10–30 million, while in most of the NL scenarios the expected increases in costs would be considerably higher. (Note: the DK data source that was excluded from the current paper is a study where the true incidence was calculated based on disease risks in returning Swedish travelers [22]. As argued in [4], the estimated increases in the number of reported cases based on this data source were unrealistically high.)

In the present study it is assumed that all reported cases are associated with some degree of clinical signs and therefore also with potential costs. Reported cases of human *Salmonella* infection may include both clinical cases and subclinical cases as healthy people may be tested for example when contact tracing. However, at least in SE, a vast majority of reported cases show clinical symptoms. Thus, it was regarded as reasonable to use reported cases as inputs in the calculations of the true number of salmonellosis cases.

### Estimations of the true number of domestic salmonellosis cases and associated costs

The estimations of the increases in the true number of cases in the various scenarios were based on a multiplier that was calculated using simulations, based on expert opinion and on the only previous study where a SE multiplier was estimated [5]. Studies from other countries have estimated higher multipliers [23], similar ones [24] as well as lower ones[25]. Using any of these would imply different estimates for the changes in the true number of cases, and, as a consequence, different cost estimates. Changing the multiplier by a certain percentage would entail changing the estimated mean costs for domestic salmonellosis cases and sequelae with the same percentage. However, underdiagosis and underreporting vary between countries [5], [22], [26], [27], and therefore country specific multipliers should be used. The multiplier estimated in the current study is the only available *Salmonella*-specific multiplier in SE based on multiple sources (including the data from [26]), and it is therefore considered to be the most accurate estimate available.

In the cost calculations, only IBS and ReA were included as sequelae to salmonellosis. Other studies have found evidence of other sequelae as well, including Inflammatory Bowel Disease [28] and meningitis [29], which implies that costs for sequelae are likely being underestimated in the current study. However, at least in the case of IBD, the number of cases triggered by salmonellosis seem to be so few that including them should not change the results noticeably [30].

Due to a lack of consistent estimates in the literature, the costs for salmonellosis and sequelae do not include VSC estimations. These costs could potentially constitute a major part of the total costs [31], and may thus lead to a considerable underestimation of the costs. The included VSL estimations were based on the transport sector in Sweden and were then transferred to the food safety sector. This kind of transfer across sectors is not unproblematic, and might lead to under- as well as overestimated costs [32]. One problem is that the sample characteristics (for example age and sex) may be different in the two studies involved in the transfer. To reduce this part of the problem, VSLY was first calculated based on the sample characteristics used in the transport sector study. This value was then, secondly, adapted to the life expectancy (adjusted for age and sex) that apply specifically to fatal SE salmonellosis cases. Thus the fact that most of the fatal cases in SE were comparatively old was accounted for in the calculations. Furthermore, a sensitivity analysis of the VSL value was made. These estimations indicate that reducing (or increasing) the VSL value by 50 percent would reduce (or increase) the costs for domestic salmonellosis cases and sequelae by 9 and 13 percent using the HCM and FCM methods, respectively. Neither of these changes would thus qualitatively change any of the net effects in Table 4.

To estimate indirect costs includes calculating the net salaries for an employee during the production loss period. Generally, production losses accrue as long as no replacement for the absent employee can be found. According to neoclassical labour market theory, there can be no involuntary unemployment in a society, and thus there will be no replacement opportunity for a company however long the sick leave. Thus, in line with this theory (the human capital approach) production losses accrue as long as the sick leave persists (until retirement). The alternative approach, called the friction cost approach, assumes that wages are not completely flexible and, as a consequence, a limited pool of involuntary unemployment does exist [33]. Accordingly a company will be able to replace an individual with a long sick leave after a certain period of search, called the friction period. For shorter periods of absence (shorter than the friction period), the two approaches generate identical estimations of indirect costs. For longer periods of illness (or even deaths), however, the human capital approach will yield higher estimates of production losses than the friction approach. In this paper both methods have been used for comparative purposes. In general, the costs using the human capital approach have generated higher estimates of indirect costs for 1) outcome class 4 of salmonellosis and 2) outcome classes 2 and 3 of IBS.

### Calculation of costs and benefits of alternative control strategies

The costs for surveillance and control in different animal species must be assessed together. The current Swedish control includes all animal species and it is reasonable to assess the scenarios with this approach as well. The *Salmonella* prevalence in one domestic species will affect the prevalence in other species and the environment. For this reason, this study aims to assess the overall situation and how it affects the prevalence and consequent health costs in humans. Domestic cases in humans may stem from sources other than domestic animals and the effect of control in animals may thus be overestimated. However, controlling *Salmonella* in domestic animals is expected to affect other sources of the infection that often have their origin in domestic animals.

The calculated costs for the current control of *Salmonella* in feed, animal production and food only include direct costs. Indirect costs would include *e.g*. those arising in the dairy or meat industry due to supply shortages caused by restrictions on infected farms or hygiene measures on restricted farms required for milk collection trucks etc. Moreover, only documented costs for on-farm eradication could be included. Thus, costs for extra working hours that could not be documented, or losses due to the need to replace old but functional buildings that could not be properly cleaned as well as other non-refundable costs were not included. This may lead to an underestimation of the costs for the current *Salmonella* control. However, trying to include indirect costs may lead to an overestimation of the control costs, as some of those costs are associated with a future improvement in the production or other economic benefits.

The estimated benefits of introducing alternative strategies for *Salmonella* surveillance in the DK and NL scenarios were also based on direct costs. Any indirect costs in these scenarios were impossible to predict. However, the DK and NL scenarios only include surveillance costs and not heat treatment of poultry feed. Administrative costs for the industry's surveillance programmes were not included either, as they were hard to predict. Thus some of the expenses in the scenarios are underestimated (heat treatment of poultry feed is mandatory in the EU and the surveillance programmes in the scenarios would entail some administrative costs) and consequently the net benefit is overestimated. The surveillance figures are based on calculations of the number of samples that would be taken in the SE animal population according to the strategies in DK and NL, respectively. Substantial changes in the SE animal population would make these estimates less reliable, but it would also affect the cost for the current programme (which is based on the current animal population size). Thus the comparison between the current control and the DK and NL scenarios would still be useful and the estimated benefits would most likely not be substantially affected. Cost for measures taken in positive herds were not included in the scenarios. These costs are difficult to estimate as they depend on the number of positive farms and on the measures taken by each farm. Losses due to restrictions in dairy herds were included in the estimate for indirect costs for *S*. Dublin in cattle herds. In pig herds, losses due to restrictions would perhaps be larger.

Potential benefits in terms of consumer and producer surplus changes at the retail stage due to a reduced cost for meat were not included in the study. If retailers face lower marginal costs due to lower input prices, the supply curve would shift outwards which would yield increases in both consumer and producer surpluses. As illustrated in Table 1, however, neither cattle farmers nor poultry farmers would face any decrease in variable costs as a result of exchanging the current Salmonella control programme. The reason for this is that lower feed prices are offset by a higher prevalence of *Salmonella* Dublin and by existing EU requirements according to the microbiologic criteria. There is therefore no reason to expect that retailers' supply curves would shift for these types of meat more than marginally as a result of exchanging the control programme. For pig farmers it is possible that marginal costs would decrease, at least in the NL scenario where surveillance costs are expected to be quite low. Although rather small (as compared to the costs of exchanging control programmes as presented in Table 4), the precise size of these reductions are not possible to predict, since the reduced costs for feed producers that benefit pig farmers specifically was not possible to extract from the data. However, price transmissions for both pork and beef have been shown to be both small and asymmetric in the Swedish food chain, which means that price decreases in earlier stages will only have a limited impact on prices in later stages [34]. We would thus expect input price changes to have a very limited impact in the retail stage, and the potential effects on consumer and producer surplus are therefore expected to be small. For these reasons, not including these potential benefits are not expected to have any significant effect on the total estimated benefits.

As the aim of this study was not to design an optimized alternative control strategy for SE, different scenarios for control of *Salmonella* in an alternative strategy were not included. The current control programme is approved by the EU and constitutes the basis for the SE additional guarantees for *Salmonella* in live animals, raw feed materials and food items. It is extremely difficult to predict the changes in import patterns as regards all of these products, but the general belief is that the import of food items would increase dramatically and immediately, and that the *Salmonella* prevalence in food items covered by the SE additional guarantees would also be expected to increase immediately. Moreover, if the feed control was abandoned, an immediate increase in *Salmonella* in pigs is to be expected. The change in cattle would be slower and perhaps alleviated by voluntary measures by the farmers. Moreover, as *S*. Dublin is already present in the Swedish cattle population (although at a lower level), some losses due to this infection are already present. Again, this is difficult to assess and thus it was decided to use figures that were published and based on official calculations by the Swedish Board of Agriculture, but to include a reduced estimate for these losses as well. The laboratory costs in all scenarios were estimated based on current prices (that include postage) and some discounts for large volumes. These prices may have been slightly over- or underestimated but there is already a market in Sweden for large numbers of *Salmonella* analyses, paid for with private as well as government money, and the current prices are thought to reflect this situation. Reducing the laboratory prices only had a large effect on the estimate for surveillance in pigs in the DK scenarios. However, these reduced estimates may not be realistic as they (as well as the original estimates) do not include sampling costs. The calculations of the current costs include costs for sampling and for sending the samples to the laboratory.

The calculations of the costs of ceasing the current control of cattle include indirect costs and are therefore not directly comparable with the costs for the control (see above). The calculations are, however, highly relevant for the SE situation and the comparison with health benefits in humans is considered valid. Thus it was regarded as more valuable to keep the indirect costs in the estimates than to exclude them. However, halving this estimate had the largest effect on the final cost for the alternative surveillance in both the DK and NL scenario.

Although the estimated benefits of ceasing the current *Salmonella* control in animal production may have been underestimated, the size of the increased costs for human illness is by far greater than the expected size of this underestimation. A higher estimate for the cost of the current control would not be expected to change the main result that it is favourable to keep the current control.

## Supporting Information

Appendix S1 Calculation of costs for human cases of salmonellosis.
(DOCX)

Appendix S2 Variables used in the Monte Carlo simulations to estimate costs for the true number of human domestic salmonellosis cases.
(DOCX)

Appendix S3 Variables used in the Monte Carlo simulations to calculate the number of Salmonella-related Reactive Arthritis (ReA) cases.
(DOCX)

Appendix S4 Variables used in the Monte Carlo simulations to calculate the number of Salmonella-related IBS cases.
(DOCX)

Appendix S5 Data sources used to fit a beta distribution for the proportion of salmonellosis cases with bloody diarrhea.
(DOCX)
