# Supplementary material for: Economic Effects of Introducing Alternative Salmonella Control Strategies in Sweden
Source: PLoS One. 2014 May 15;9(5):e96446. doi: 10.1371/journal.pone.0096446 (PMC4022667; doi:10.1371/journal.pone.0096446)
Supplement: Appendix S1 — Calculation of costs for human cases of salmonellosis. (DOCX) [file pone.0096446.s001.docx]

Table S1a: Variable used in the Monte Carlo-simulations to estimate probabilities of under reporting in the reporting pyramid and the outcome tree for salmonellosis: notation, estimates and sources

| Notation | Variable | Distribution/ Point estimate | Sources |
| --- | --- | --- | --- |
| *A* | Probability of consulting a GP if having a bloody diarrhoea | BetaPert(0.61; 0.95; 0.99; 4) | 0.61: [[1](#_ENREF_1)], 0.95: Expert opinion *, 0.99:Expert opinion * |
| *B* | Probability of having to submit a stool sample at a GP if having bloody diarrhoea | BetaPert(0.39; 0.95; 0.99; 4) | 0.39: [[1](#_ENREF_1)], 0.95: Expert opinion *, 0.99:Expert opinion * |
| *C* | Probability of consulting a GP if having non-bloody diarrhoea | BetaPert(0.035; 0.08; 0.08; 4) | 0.035: Expert opinion * , 0.08: [[1](#_ENREF_1)] |
| *D* | Probability, given that a GP consultation is made, of having to submit a stool sample at a GP when having non-bloody diarrhoea | BetaPert(0.48; 0.5; 0.75; 4) | 0.48: [[1](#_ENREF_1)], 0.5: Expert opinion *, 0.75: Expert opinion * |
| *E* | Probability of having to submit a stool sample if hospitalized | BetaPert(0.84; 0.95; 0.99; 4) | 0.84: [[1](#_ENREF_1)], 0.95: Expert opinion *, 0.99: Expert opinion * |
| *F* | Probability that a positive test result gets reported | 0.999 | Expert opinion *** |
| *G* | Probability of submitted stool sample being analysed for *Salmonella* bacteria (GP) | 0.99 | Expert opinion ** |
| *H* | Probability of submitted stool sample being analysed for *Salmonella* bacteria (hospital) | 0.99 | Expert opinion ** |
| *I* | Probability of a positive test result if patient is infected | BetaPert(0.7; 0.8; 0.8; 4) | 0.7: Expert opinion **, 0.8: Expert opinion ** |
| *J* | Probability of getting bloody diarrhoea if having salmonellosis | Beta (2.06; 3.78) | Fit distribution, see Appendix B for details |
| *K* | Probability of death due to salmonellosis | BetaPert(0.0044; 0.04; 0.04;4) | 0.0044: [[2](#_ENREF_2)]**** , 0.04: [[3](#_ENREF_3)] |
| *L* | Number of reported salmonellosis cases | Different point estimates | Point estimate specific for each scenario according to Table 2 |
| *M* | Number of reported hospitalized salmonellosis cases | 449/3939*L | 449:[[4](#_ENREF_4)] for 2006 , 3939:[[5](#_ENREF_5)], mean for 2005-2008 |

* Based on four expert estimates:three County Medical Officers with long experience of infectious diseases and one epidemiologist from Smittskyddsinstitutet (SMI), with an extensive experience of gastrointestinal diseases.

** Based on two expert estimates: One chief microbiologist at the reference laboratory for EHEC, SMI and one epidemiologist from SMI, with an extensive experience of gastrointestinal diseases.

*** Based on one expert estimate: An epidemiologist from SMI, with an extensive experience of gastrointestinal diseases.

**** Mean yearly number with salmonellosis as main death cause (2000-09) / simulated number of cases in population

Table S1b: Model used to estimate probabilities of underreporting in the reporting pyramid and the outcome tree for salmonellosis in the Monte Carlo-simulations, probabilities for those visiting a GP or being hospitalized

| Probabilities | GP | Hospitalized |
| --- | --- | --- |
| Sample taken | *J***B* + (1-*J*)**D* | *E* |
| Sample analysed for *Salmonella* | *G* | *H* |
| Positive test result | *I* | *I* |
| Result reported | *F* | *F* |
| Probability that a case gets reported | (*J***B* + (1-*J*)**D*)**G***I***F* | *E*H*I*F* |
| Real number of cases in population that seek care | (l-*M*)/ ((*J***B* + (1-*J*)**D*)**G***I***F*) (called *RGP* below) | *M/*(*E*H*I*F*) (called *RH* below) |

The probability of visiting a GP if you get salmonellosis is given by:

*J***A* + (1-*J*)**C*

which implies that the true total number of cases in the population (including those that do not seek care) can be calculated as:

(*RGP*+*RH*)/(*J***A* + (1-*J*)**C*)
(called *RT* below)

The number of cases in the four different outcome classes can then be estimated as described in Table S1c.

Table S1c: Model used to calculate the true number of human domestic salmonellosis cases in the different outcome classes

| Outcome class | Number of cases |
| --- | --- |
| Outcome class 1 (no care) | *RT*-(*RGP*+*RH*) |
| Outcome class 2 (GP only) | *RGP* |
| Outcome class 3 (GP and hospital) | *RH*-*RT***K* |
| Outcome class 4 (death) | *RT***K* |

**References**

(1) Haagsma J, Geenen P, Ethelberg S, Fetsch A, Hansdotter F, et al. (2012) Community incidence of pathogen-specific gastroenteritis: reconstructing the surveillance pyramid for seven pathogens in seven European Union member states. Epidemiology and Infection 1: 1-15.

(2) Socialstyrelsen National Death Registry.

(3) Mead PS, Slutsker L, Dietz V, McCaig LF, Bresee JS, et al. (1999) Food-related illness and death in the United States. Emerging Infectious Diseases 5: 607-625.

(4) Socialstyrelsen National patient registry.

(5) Smittskyddsinstitutet SmiNet, database of registered salmonellosis cases.
