# Supplementary material for: Economic Effects of Introducing Alternative Salmonella Control Strategies in Sweden
Source: PLoS One. 2014 May 15;9(5):e96446. doi: 10.1371/journal.pone.0096446 (PMC4022667; doi:10.1371/journal.pone.0096446)
Supplement: Appendix S2 — Variables used in the Monte Carlo simulations to estimate costs for the true number of human domestic salmonellosis cases. (DOCX) [file pone.0096446.s002.docx]

| Variable | Distribution/ Point estimate | Sources |
| --- | --- | --- |
| Hospitalized patients, Mean duration / visit | 4.92 | [[1](#_ENREF_1)] |
| Number of visits for all hospitalized patients | 468 | [[1](#_ENREF_1)] |
| Number of hospitalized patients | 449 | [[1](#_ENREF_1)] |
| Hospitalized patients, Mean duration / patient | 5.13 | Calculation* |
| Mean illness duration, days, outcome class 1 (no care) | 3.36 | [[2](#_ENREF_2)]** |
| Mean illness duration, outcome class 2 (GP only) | 11.44 | [[2](#_ENREF_2)]*** |
| Mean illness duration, outcome class 3 (hospitalization) | 16.57 | Calculation**** |
| Proportion of salmonellosis cases who are children (0-11 years old) | 0.1482 | Based on data from [[3](#_ENREF_3)] |
| Average age of children (0-11 years old) with salmonellosis | 4.3 | Calculations based on [[3](#_ENREF_3)] |
| Cost / GP consultation (€) | 196 | [[4](#_ENREF_4)] |
| Cost / hospitalization (€) | 3133 | [[4](#_ENREF_4)] |
| Transport costs /case (€) | 6 | [[5](#_ENREF_5)]***** |
| Medication costs/case (€) | 1 | [[5](#_ENREF_5)]****** |
| Proportion of parents’ allowance registered by women | 63 % | [[6](#_ENREF_6)] |
| Value of a Statistical Life (VSL), €, 2006 | 2464788 | [[7](#_ENREF_7)] |
| Value of a Statistical Life-Year (VSLY), € | 65694 | Calculations based on [[7](#_ENREF_7)] |
| Intangible costs per death, € | 875529 | Calculations based on [[7](#_ENREF_7)] and [[8](#_ENREF_8)] |
| Average production loss per death, human capital approach, € | 140845 | Calculations based on [[9-11](#_ENREF_9)] |

* “Mean duration/visit” * “number of visits for hospitalized patients” / “number of hospitalized patients”: 4.92*468/449

** p 408, Table 4.7b, average from ”responders’ mean” and ”overall mean”

*** p 410-411, average from ”responders’ mean” and ”overall mean”

**** “Mean illness duration, outcome class 2” + “Hospitalized patients
Mean duration / patient”: 11.44 + 5.13

***** Cost updated using Consumer Price Index (CPI) for transports from 1998 to 2009

****** Cost updated using CPI for medication from 1998 to 2009

**References**

(1) Socialstyrelsen National patient registry.

(2) FSA (2000) A Report of the Study of Infectious Intestinal Disease in England. London: Food Standard Agency.

(3) Smittskyddsinstitutet SmiNet, database of registered salmonellosis cases.

(4) SKL KPP (Cost Per Patient) database.

(5) SLV (1999) Mat Upp - intensivstudie av matförgiftningar i Uppsala kommun under ett år. SLV.

(6) Eriksson H (2009) Är det verkligen ekonomin som styr? En studie av ekonomiska drivkrafter vid vård av barn. Working Papers in Social Insurance, Försäkringskassan.

(7) SIKA (2008) SIKA PM 2008:3 Samhällsekonomiska principer och kalkylvärden för transportsektorn: ASEK 4.

(8) Pires SM, L. dK and T. H (2011) Estimation of the relative contribution of different food and animal sources to human Salmonella infections in the European Union, Scientific report/Technical report submitted to EFSA. Denmark: National Food Institute, Technical University of Denmark.

(9) SCB Data on salaries including social benefits.

(10) SCB Data on life length and survival probabilities in Sweden.

(11) Socialstyrelsen National Death Registry.
