# Supplementary material for: Economic Effects of Introducing Alternative Salmonella Control Strategies in Sweden
Source: PLoS One. 2014 May 15;9(5):e96446. doi: 10.1371/journal.pone.0096446 (PMC4022667; doi:10.1371/journal.pone.0096446)
Supplement: Appendix S3 — Variables used in the Monte Carlo simulations to calculate the number of Salmonella-related Reactive Arthritis (ReA) cases. (DOCX) [file pone.0096446.s003.docx]

| Variable | Distribution/ Point estimate | Sources |
| --- | --- | --- |
| Proportion of salmonellosis cases that develops ReA | BetaPert(0.0230; 0.08; 0.15; 4) | [[1](#_ENREF_1)] |
| Proportion of ReA cases that consults a GP | Beta(10; 37) | [[2](#_ENREF_2)] |
| Proportion of ReA cases who are hospitalized | Beta(2; 45) | [[2](#_ENREF_2)] |
| Number of sick leave days | BetaPert(26; 26; 53; 4) | [[3](#_ENREF_3), [4](#_ENREF_4)] |
| Average age of children (0-11 years old) who develop ReA | 4.3 | See corresponding variable for salmonellosis |
| Costs/patient for GP consultation(s) (€) | 1467 | Own calculations based on [[4](#_ENREF_4)] |
| Cost/patient for hospitalization (€) | 6373 | Own calculations based on [[4](#_ENREF_4)] |

**References**

(1) Raybourne RB, Williams KM and T. R (2003) Food poisoning: economic implications. Encyclopedia of food sciences and nutrition. London: : Elsevier Science Ltd. pp. 2672-2682.

(2) Hannu T, Mattila L, Rautelin H, Pelkonen P, Lahdenne P, et al. (2002) Campylobacter-triggered reactive arthritis: a population-based study. Rheumatology 41: 312-318.

(3) Mangen MJ, de Wit G and Havelaar AH (2003) Campylobacteriosis in the Netherlands: Estimating the cost-of-illness and the disease burden. IJMM International Journal of Medical Microbiology 293: 27.

(4) Soderlin MK, Kautiainen H, Jonsson D, Skogh T and Leirisalo-Repo M (2003) The costs of early inflammatory joint disease: a population-based study in southern Sweden. Scandinavian Journal of Rheumatology 32: 216-224.
