# Supplementary material for: Economic Effects of Introducing Alternative Salmonella Control Strategies in Sweden
Source: PLoS One. 2014 May 15;9(5):e96446. doi: 10.1371/journal.pone.0096446 (PMC4022667; doi:10.1371/journal.pone.0096446)
Supplement: Appendix S4 — Variables used in the Monte Carlo simulations to calculate the number of Salmonella-related IBS cases. (DOCX) [file pone.0096446.s004.docx]

| Variable | Distribution/ Point estimate | Sources |
| --- | --- | --- |
| Proportion of salmonellosis cases that develops IBS | Weighted (AR)* | [[1-5](#_ENREF_1)] |
| Proportion of IBS cases consulting a GP | 0.32 | [[6](#_ENREF_6), [7](#_ENREF_7)] |
| Proportion of ReA cases who gets hospitalized | 0.19 | [[7](#_ENREF_7)] |
| Illness duration, years | BetaPert(0.5;1; 6; 4) | Based on data from [[8-13](#_ENREF_8)] |
| Average age of children (0-11) who develop IBS | 4.3 | See corresponding variable for salmonellosis |
| Costs/patient for GP consultation(s) (€) | 343 | [[14](#_ENREF_14)], mean from 2008-09 |
| Cost/patient for hospitalization (€) | 3044 | [[14](#_ENREF_14)], mean from 2006-09 |
| Transport costs per patient (€) | 7 | [[15](#_ENREF_15)]** |

* AR=Attributable Risk. Based on beta distributions calculated from the sources

** Updated using Consumer Price Index for transport 1998-2009

**References**

(1) Mearin F, Perez-Oliveras M, Perello A, Vinyet J, Ibanez A, et al. (2005) Dyspepsia and irritable bowel syndrome after a Salmonella gastroenteritis outbreak: One-year follow-up cohort study. Gastroenterology 129: 98-104.

(2) Parry SD, Stansfield R, Jelley D, Gregory W, Phillips E, et al. (2003) Does bacterial gastroenteritis predispose people to functional gastrointestinal disorders? A prospective, community-based, case-control study. American Journal of Gastroenterology 98: 1970-1975.

(3) Jung IS, Kim HS, Park H and Lee SI (2009) The Clinical Course of Postinfectious Irritable Bowel Syndrome A Five-year Follow-up Study. Journal of Clinical Gastroenterology 43: 534-540.

(4) Rodriguez LAG and Ruigomez A (1999) Increased risk of irritable bowel syndrome after bacterial gastroenteritis: cohort study. Br Med J 318: 565-566.

(5) Wang LH, Fang XC and Pan GZ (2004) Bacillary dysentery as a causative factor of irritable bowel syndrome and its pathogenesis (vol 53, pg 1096, 2004). Gut 53: 1390-1390.

(6) Osterberg E, Blomquist L, Krakau I, Weinryb RM, Asberg M, et al. (2000) A population study on irritable bowel syndrome and mental health. Scandinavian Journal of Gastroenterology 35: 264-268.

(7) Hahn BA, Kirchdoerfer LJ, Fullerton S and Mayer E (1997) Patient-perceived severity of irritable bowel syndrome in relation to symptoms, health resource utilization and quality of life. Alimentary Pharmacology & Therapeutics 11: 553-559.

(8) Gwee KA, Graham JC, McKendrick MW, Collins SM, Marshall JS, et al. (1996) Psychometric scores and persistence of irritable bowel after infectious diarrhoea. Lancet 347: 150-153.

(9) Ji S, Park H, Lee D, Song YK, Choi JP, et al. (2005) Post-infectious irritable bowel syndrome in patients with Shigella infection. Journal of Gastroenterology and Hepatology 20: 381-386.

(10) Kim HS KM, Ji SW, Park H. (2006) The development of irritable bowel syndrome after Shigella infection: 3 year follow-up study. Korean J Gastroenterol 47: 300-305.

(11) Marshall JK, Thabane M, Garg AX, Clark WF, Salvadori M, et al. (2006) Incidence and epidemiology of irritable bowel syndrome after a large waterborne outbreak of bacterial dysentery. Gastroenterology 131: 445-450.

(12) McKeown ES, Parry SD, Stansfield R, Barton JR and Welfare MR (2006) Postinfectious irritable bowel syndrome may occur after non-gastrointestinal and intestinal infection. Neurogastroenterology and Motility 18: 839-843.

(13) Neal KR, Barker L and Spiller RC (2002) Prognosis in post-infective irritable bowel syndrome: a six year follow up study. Gut 51: 410-413.

(14) Sundström K (2011) Samhällskostnader för salmonellos, campylobacterios och EHEC [Societal costs for salmonellosis, campylobacteriosis and EHEC].

(15) SLV (1999) Mat Upp - intensivstudie av matförgiftningar i Uppsala kommun under ett år. SLV.
