# Supplementary material for: Economic Effects of Introducing Alternative Salmonella Control Strategies in Sweden
Source: PLoS One. 2014 May 15;9(5):e96446. doi: 10.1371/journal.pone.0096446 (PMC4022667; doi:10.1371/journal.pone.0096446)
Supplement: Appendix S5 — Data sources used to fit a beta distribution for the proportion of salmonellosis cases with bloody diarrhea. (DOCX) [file pone.0096446.s005.docx]

| % bloody diarrhoea | Country | Reference |
| --- | --- | --- |
| 10-65 | USA | [[1-10](#_ENREF_1)] |
| 23 | USA/ Canada | [[11](#_ENREF_11)] |
| 56 | the Netherlands | [[12](#_ENREF_12)] |
| 7 | Australia | [[13](#_ENREF_13)] |
| 18 and 22 | Sweden | Personal communication with SMI* |

* Smittskyddsinstitutet (Swedish Institute for Communicable Disease Control)

**References**

(1) Anonymous (2007) Multistate Outbreak of Salmonella Typhimurium Infections Associated with Eating Ground Beef -- United States, 2004. Morbidity and Mortality Weekly Report. pp. 180-182.

(2) Morgan O, Milne L, Kumar S, Murray D, Man W, et al. (2007) Outbreak of Salmonella Enteritidis phage type 13a: case-control investigation in Hertsmere, United Kingdom. Euro surveillance: bulletin européen sur les maladies transmissibles= European communicable disease bulletin 12: E9.

(3) Anonymous (2004) Salmonella Serotype Typhimurium Outbreak Associated with Commercially Processed Egg Salad. Morbidity and Mortality Weekly Report. pp. 1132-1134.

(4) Anonymous (2008) Multistate Outbreak of Human Salmonella Infec- tions Associated with Exposure to Turtles --- United States, 2007–2008. Morbidity and Mortality Weekly Report. pp. 69–72.

(5) Anonymous (2003) Multistate Outbreak of Salmonella Typhimurium Infections Associated with Drinking Unpasteurized Milk---Illinois, Indiana, Ohio and Tennessee 2002-2003, Morbidity and Mortality Weekly Report 52(26), p.613-615. pp. 613-615.

(6) Anonymous (1999) Outbreak of Salmonella Serotype Muenchen Infections Associated with Unpasteurized Orange juice -- United States and Canada, June 1999, Morbidity and Mortality Weekly Report, 1999. pp. 582-585.

(7) Anonymous (2007) Three Outbreaks of Salmonellosis Associated with Baby Poultry from Three Hatcheries --- United States, 2006, Morbidity and Mortality Weekly Report, 2007. pp. 273-276.

(8) Anonymous (1997) Multidrug-Resistant Salmonella Serotype Typhimurium -- United States, 1996, Morbidity and Mortality Weekly report, 1997. pp. 308-310.

(9) Anonymous (1995) Outbreak of Salmonella Serotype Typhimurium Infection Associated with eating Raw Ground Beef -- Wisconsin, 1994, Morbidity and Mortality Weekly Report. pp. 905-909.

(10) Anonymous (2007) Salmonella Serotype Enteritidis Infections Among Workers Producing Poultry Vaccine --- Maine, November-December 2006, Morbidity and Mortality Weekly Report, 2007. pp. 877-879.

(11) Anonymous (2007) Salmonella Oranienburg Infections Associated with Fruit Salad Served in Health-Care Facilities --- Northeastern United States and Canada, 2006, Morbidity and Mortality Weekly Report, 2007. pp. 1025-1028.

(12) van Duynhoven Y, Widdowson MA, de Jager CM, Fernandes T, Neppelenbroek S, et al. (2002) Salmonella enterica serotype enteritidis phage type 4b outbreak associated with bean sprouts. Emerging Infectious Diseases 8: 440-443.

(13) Moffatt CRM, Combs BG, Mwanri L, Holland R, Delroy B, et al. (2006) An outbreak of Salmonella Typhimurium phage type 64 gastroenteritis linked to catered luncheons in Adelaide, South Australia, June 2005. Communicable Diseases Intelligence 30: 443-448.
